# A review on the *Scorpaena plumieri* fish venom and its bioactive compounds

**DOI:** 10.1186/s40409-016-0090-7

**Published:** 2016-12-21

**Authors:** Fabiana V. Campos, Thiago N. Menezes, Pedro F. Malacarne, Fábio L. S. Costa, Gustavo B. Naumann, Helena L. Gomes, Suely G. Figueiredo

**Affiliations:** 1 Departamento de Ciências Fisiológicas, Centro de Ciências da Saúde, Universidade Federal do Espírito Santo, Av. Marechal Campos 1468, 29040-090 Vitória, ES Brazil; 2 Departamento de Bioquímica e Imunologia, Instituto de Ciências Fisiológicas, Universidade Federal de Minas Gerais, Belo Horizonte, MG Brazil; 3 Diretoria do Centro de Pesquisa e Desenvolvimento, Fundação Ezequiel Dias, Belo Horizonte, MG Brazil

**Keywords:** Scorpionfish, *Scorpaena plumieri* venom, Inflammatory response, Proteolytic activity, Cardiovascular activity, Sp-GP, Plumieribetin, C-type lectins, Sp-CTx

## Abstract

The most poisonous fish species found along the Brazilian coast is the spotted scorpionfish *Scorpaena plumieri*. Though hardly ever life-threatening to humans, envenomation by *S. plumieri* can be quite hazardous, provoking extreme pain and imposing significant socioeconomic costs, as the victims may require days to weeks to recover from their injuries. In this review we will walk the reader through the biological features that distinguish this species as well as the current epidemiological knowledge related to the envenomation and its consequences. But above all, we will discuss the challenges involved in the biochemical characterization of the *S. plumieri* venom and its compounds, focusing then on the successful isolation and pharmacological analysis of some of the bioactive molecules responsible for the effects observed upon envenomation as well as on experimental models. Despite the achievement of considerable progress, much remains to be done, particularly in relation to the non-proteinaceous components of the venom. Therefore, further studies are necessary in order to provide a more complete picture of the venom’s chemical composition and physiological effects. Given that fish venoms remain considerably less studied when compared to terrestrial venoms, the exploration of their full potential opens a myriad of possibilities for the development of new drug leads and tools for elucidating the complex physiological processes.

## Background

**Fig. 1:**
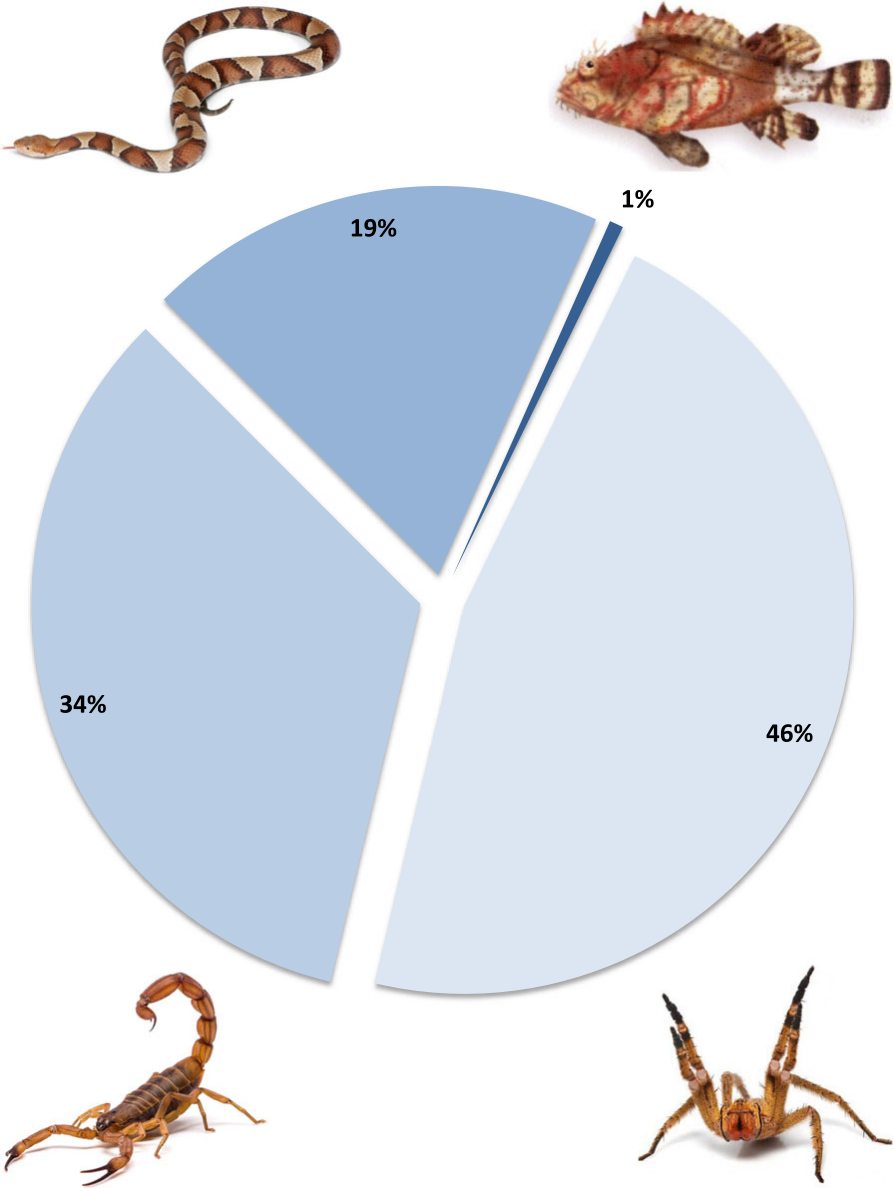
Fish venoms in the literature. Comparison between the number of entries (%) obtained through a search for sequences of bioactive proteins from spiders, scorpions, snakes and fish in the UniProtKB database. Entry terms: spider/scorpion/snake/fish: organism; toxins: keyword

The immense pharmacological potential contained in the venoms of several species throughout the globe has been profoundly remarked upon and — in relation to terrestrial animals — considerably well explored. On the other hand, marine and aquatic animals remain relatively underrepresented in the literature [1–3]. A search in the UniProtKB databank reveals a large number of entries for scorpion, spider and snake protein toxins, while data on marine and aquatic animals — particularly fish — remain rather scarce (Fig. 1). This discrepancy can be somewhat explained by the fact that fish do not seem to pose as large a threat from an epidemiological point of view [1]. Moreover, the extreme lability of the toxic components combined with the challenges involved in extracting, isolating and storing the venom makes their study and exploration a task that only the most tenacious researchers can perform [1, 4, 5]. Nonetheless, fish comprise more than half of all venomous vertebrates [6, 7], so much so that a phylogenetic analysis conducted by Smith and Wheeler in 2006 [6] suggests that up to 1,200 fishes in 12 clades should be assumed venomous. Thus, an effort towards a deeper understanding of fish venoms contributes not only to the discovery of new drug leads but also to a more efficient exploration of our biodiversity.

The Brazilian coast is home to a large variety of venomous fish species, the most poisonous being the spotted scorpionfish *Scorpaena plumieri* [8–11]. It is noteworthy that the Scorpaeniformes (families Scorpaenidae and Synanceiidae) are the most venomous marine fishes in the world [11, 12].

**Fig. 2:**
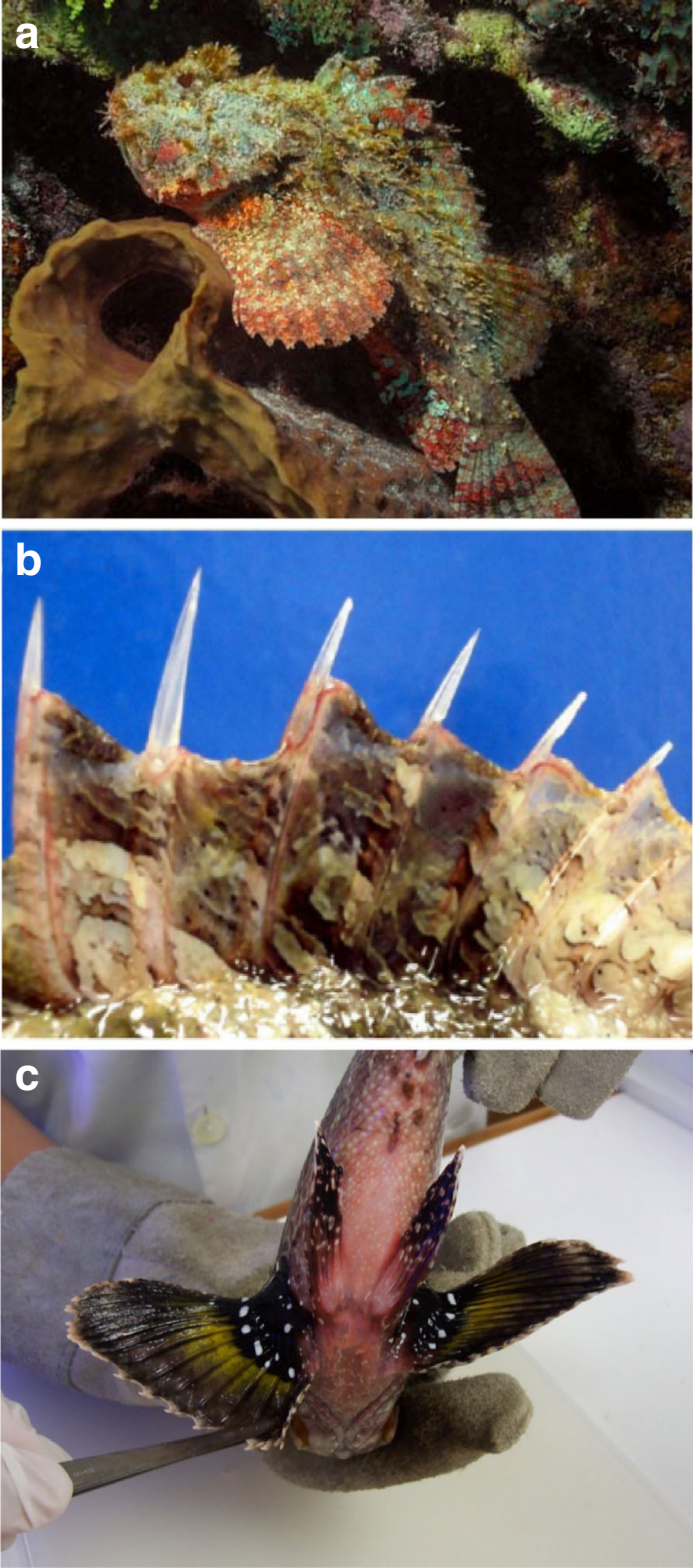
The spotted scorpionfish *Scorpaena plumieri*. **a** Picture of a specimen of *S. plumieri* highlighting its camouflage capability. **b** Erected dorsal spines covered in mucous skin form — along with the pelvic and anal fin spines — the venom apparatus of *S. plumieri*. **c** White spots against a black background on the inner part of the pectoral fins, a characteristic feature of this species

*S. plumieri* Bloch, 1789, commonly known in Brazil as *mangangá*, *niquim-de-pedra* or *mamangava* [11], can be found along the Brazilian southeastern coast, as well as off Florida, in the Gulf of Mexico, the Caribbean, the Bahamas and Bermudas. It usually dwells in shallow waters and reefs, remaining motionless and disguised among rocks and plants [13]. This camouflaging capability is paramount in order to ambush prey and to mislead predators (Fig. 2a). Like other scorpionfishes, the representatives of this species are fairly large (up to 50 cm), with 12 dorsal, 2 pelvic and 3 anal short and thick fin spines (Fig. 2b) covered with mucous-rich integumentary sheath [14]. The identification of the specimens is made through the observation of white spots or blotches on a black background on the inner portion of the pectoral fins [15] (Fig. 2c).

The venom gland in scorpionfishes is not a well-defined structure, but consists of a group of secretory cells lying within the spines anterolateral grooves, without an excretory duct [11, 16]. Therefore, the venom apparatus in this species comprises the spines plus the integumentary sheath associated with them. Envenomation occurs through mechanical pressure on the spines, which tears the integumentary sheath to allow the release of the venom along with the mucus present in the skin [17, 18]. This quite primitive venomous apparatus, common among poisonous fishes, has evolved mostly for defensive purposes, which is consistent with its involuntary delivery mechanism [1, 2].

Humans can become victims of *S. plumieri* when fishermen, divers and bathers accidentally tread on or handle the fish and have their skin perforated by the spines [11]. The clinical manifestations of accidents include local and systemic effects. The first symptom is always excruciating pain, followed by edema, erythema, occasional skin necrosis, adenopathy, nausea, vomiting, agitation, malaise, sweating, diarrhea, tachycardia and arrhythmias, culminating, in some cases, in severe hypotension [11]. The treatment is symptomatic and usually consists of soaking the affected limb in hot water (45–50 °C) at least until the pain is relieved, though why such heat is effective remains under discussion [11].

Envenomation by *S. plumieri*, though hardly ever life-threatening to humans, imposes considerable socioeconomic costs, given that fishermen — the group most prone to accidents — may require days to weeks to recover from their injuries [11]. And even if accidents involving *S. plumieri* are — at least according to the official reports made to the Notifiable Diseases Information System (SINAN) — somewhat rare when compared to other venomous aquatic species found in Brazil, the potential severity of the injuries caused by these stings justifies the need for a thorough investigation of these cases [19].

The Laboratory of Protein Chemistry of the Federal University of Espírito Santo (UFES), Brazil, which has been studying the venom of *S. plumieri* for over a decade, is responsible for the great majority of the literature on this topic. Considerable progress has been made in relation to the biochemical and pharmacological properties of the crude venom extract [20–23] and a few bioactive molecules have been isolated and analysed [20, 24–28]. In this review, we will focus on the discussion of the chemical and physio-pharmacological properties of *S. plumieri* venom along with those of the bioactive molecules isolated so far.

## Extraction and chemical composition of *S. plumieri* venom

Given that the venom gland in *S. plumieri* is not a well-defined structure, the collection of the venom in an uncontaminated form is technically difficult. Hence, *S. plumieri* venom studies have been conducted using the extract from its venomous apparatus. This venomous extract (referred to as SpV) has been obtained according to the batch method [4] adapted by Carrijo et al. [20], in which an average-sized fish (15–20 cm) yields ≈ 10–16 mg of total protein.

SpV is mucous-rich, which presents a considerable challenge to its study. Nevertheless, the major hindrance to elucidating the nature of the venom has been the instability of its active components, which could be partially due to the presence of endogenous proteolytic enzymes [20, 24].

The protein complexity of SpV is evident from a number of different components found when the extract was subjected to two-dimensional SDS-PAGE. This analysis revealed about two hundred protein spots (6 to 120 kDa) with a predominance of anionic proteins [29]. A similar molecular weight range has been described for the protein components of other fish venoms [30–32].

In addition to the protein constituents, some other active compounds, such as biogenic amines have been described in fish venoms [33–37]. However, these components — which also present important implications for venom activity — have yet to be explored in *S. plumieri* venomous extract.

## Biological activities of *S. plumieri* venom extract (Spv)

Studies conducted on SpV have shown the enormous diversity and complexity of its biological activities. SpV was found to perform lethal, hemolytic, cardiovascular, inflammatory, integrin-binding-inhibitory and proteolytic activities [20, 22–24, 27, 29]. This spectrum of activities — observed in experimental animals — resembles those of other fish venoms previously described [1, 2].

The first study focusing on biological properties of SpV was reported by Carrijo et al. [20]. Intravenous injection of SpV in mice induced loss of muscular coordination, paralysis, urination, hypersalivation, convulsions and respiratory failure, followed by death. The LD_50_ was estimated to be 0.28 mg/kg, a value comparable to those reported for venoms of other scorpaeniform fish [4, 38, 39]. The venom also displays dose-dependent hemolytic activity on rabbit erythrocytes [20]. Furthermore, as SpV lacks phospholipase A2 activity — much like other fish venoms — the hemolysis can be explained by pore formation activity [25].

As previously mentioned, the first and most notable effect of the envenomation is clinically characterized by intense edema, erythema and excruciating pain, which are generally associated with an inflammatory response [11]. Experiments conducted using the mice paw test have shown that the injection of SpV into the footpad induces intense edema that is time- and dose-dependent [29]. In contrast, a pronounced nociceptive response reaches a plateau at low doses (≥15 μg/paw) [29]. This inflammatory response is characterized by a release of pivotal pro-inflammatory mediators (TNF, IL-6 and MCP-1) that may be associated with histopathological changes observed in paw tissue, distinguished by cellular infiltration of mainly neutrophils followed by mononuclear cells after 12 h [23]. SpV-induced edema was found to be significantly reduced by previous administration of a serine-protease inhibitor (aprotinin) or a bradykinin B2 receptor antagonist (icatibant), while pretreatment with a non-selective COX inhibitor (diclofenac sodium) and a H_1_ receptor antagonist (promethazine) had less effect, suggesting that the kallikrein-kinin system (KKS) plays a major role in the edema formation [23].

In addition to the local inflammatory response, a systemic reaction is triggered after SpV injection in the footpad or peritoneal cavity of mice, leading to endothelial barrier dysfunction, microvascular hyperpermeability and sustained inflammatory response, culminating in alveolar edema and neutrophilic inflammation. Alveolar macrophages (AM) and neutrophils act as a source of matrix metalloproteinases that together play a key role in the cascade of events leading to lung injury. These findings also confirm a central role for macrophage and neutrophils in the pathogenesis of venom-induced lung injury and also the importance of AMs in the resolution of this SpV-triggered process [21].

These inflammatory responses may be due to the activity of proteases, hyaluronidases and integrin-inhibiting factors that could affect the extracellular matrix (ECM). And indeed, enzymatic activities are prominently described in the literature on fish — and terrestrial — venoms [40–43]. These enzymes initiate reactions that can contribute to local and systemic effects by acting as “spreading factors”, either increasing tissue permeability and facilitating the spread of other constituents of the venom or causing direct tissue damage to the prey [44]. Furthermore, these enzymes are also involved in the post-translational processing of the many toxins in the venom [45].

SpV was shown to hydrolyse casein and gelatin [20]. These proteolytic activities were also reported in the venoms of the fish *Potamotrygon falkneri* and *Thalassophyne maculosa*, respectively [31, 32]. Akin to most fish venoms, SpV is devoid of any phospholipase activity, albeit phospholipase C activity has been detected in the *Scatophagus argus* venom [46].

Due to their pivotal role, integrins — which are receptors of the ECM — are targets for several naturally occurring toxins. There are several literature reports of these molecules in snake venoms, including desintegrins [47] and C-type lectins [48–50]. On the other hand, only recently have these molecules been reported in fish venoms. A cell-free binding assay showed that SpV inhibited the binding of integrins α1β1, α2β1, α3β1 and α7β1 to their respective ligands: collagen IV, collagen I, laminin-332 and laminin-111 [27].

Among all the effects caused by fish venoms, cardiovascular activity has been the main subject of research in the field [1, 2]. Clinical reports have shown that the symptoms of *S. plumieri* envenomation include respiratory distress and tachycardia [11]. Similarly, it was observed in animal models that SpV increases mean arterial pressure (MAP) in a dose-dependent manner. However, biphasic responses — characterized by an initial increase followed by a pronounced fall of the MAP — are achieved using higher doses (338 μg/kg), leading to the death of the animal after a few minutes [22]. This phenomenon was also observed in other fish venoms, such as *P. volitans*, *S. horrida* and *S. guttata*. The high-pressure phase has been associated with adrenoceptors while the hypotensive phase seems to involve muscarinic receptors and/or nitric oxide synthesis [51, 52].

In isolated hearts, SpV produces dose-dependent and transient positive ventricular chronotropic, inotropic and lusitropic effects. These responses are attenuated by a non-selective β-adrenergic antagonist, evidencing that the venom compounds could act — at least in part — directly through the presence of some adrenergic agonist in the venom and/or indirectly via the release of endogenous stores of norepinephrine from the sympathetic varicosities in the heart [22].

Besides the activity on the cardiac muscle, SpV also produces vascular effects. SpV induces a dose-dependent increase in perfusion pressure (CPP) on the coronary bed, and a biphasic effect on intact and pre-contracted rat aortic rings — characterized by an initial and transient relaxing phase followed by a sustained contractile phase [22, 24]. It is noteworthy that variations in vascular responses induced by the same fish venom have been observed in studies applying different experimental models [1].

The unravelling of the precise action mechanism behind all the biological activities attributed to venoms depends on the isolation of the substances responsible for each one of these activities.

**Fig. 3:**
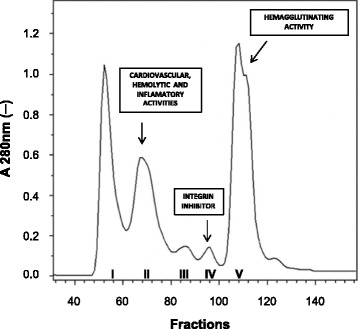
Elution profile of the gel filtration fractionation of the *Scorpaena plumieri* extract (SpV). A sample of SpV (approximately 83 mg of protein) was applied on a Sephacryl S-200 HR column (2.0 cm × 120 cm) previously equilibrated and eluted with 0.01 M phosphate buffer at pH 7.6 with 0.4 M NaCl at 4 °C. Flow rate, 5.25 mL/h, fractions of 1.75 mL. Figure adapted from [27]

An initial fractionation procedure applying gel filtration chromatography yielded five fractions from SpV [20]. This approach succeeded in separating the cardiovascular activity from the integrin inhibitory activity, though not from the hemolytic or inflammatory activities. In addition, this procedure also revealed a hemagglutinating fraction (Fig. 3). While the proteolytic and lectin-related biological activities were shown to be highly stable, a great deal of instability was shown by the hemolytic, cardiovascular and inflammatory activities [20, 24].

Finally, despite all the difficulties surrounding the purification of active proteins from fish venoms our group has isolated four proteins from SpV. In the following section we will discuss the biochemical, physiological and pharmacological features of these proteins.

## Bioactive proteins isolated from Spv

### *Scorpaena plumieri* gelatinolytic protease (Sp-GP)

The first toxin isolated from SpV was Sp-GP, a 72 kDa protease with gelatinolytic activity. In fact, to the best of our knwoledge, it was the first active protein isolated from a scorpionfish [20]. Homogeneity was reached through three purification steps: gel filtration (Fig. 3), ion exchange, and reverse-phase chromatography. The ineffectiveness of efforts at N-terminal sequencing suggests that the enzyme is N-terminally blocked. The optimal pH value for its activity was found to be within the range of 7–8 [20]. Although many fish venoms were found to perform proteolytic activity, the only other isolated fish venom proteases comprise a group of five toxins termed natterins (5.9–41.4 kDa) found in the venom of the toadfish *Thalassophryne nattereri*. These proteases cleave human kininogen and degrade type I and type IV collagen in vitro. The latter leads to direct induction of necrosis, stimulating an inflammatory response, which, in turn, correlates with the edema-inducing effects of the toxin [53, 54].

### Lectins

Extracts from vegetable or animal sources — venoms, for instance — have the ability to induce the agglutination of hemocytes and to disrupt cell-ECM interactions [48, 55]. These abilities are related to the activity of molecules with carbohydrate-binding properties: the lectins.

Two lectins — (i) plumieribetin, a lectin homologous to monocot mannose-binding B-type lectin and (ii) a group of five isolectins (Sp-CL 1–5) homologous to fish C-type lectins — were purified from *S. plumieri* venom [27, 28].

Plumieribetin was purified with a high degree of homogeneity by gel filtration chromatography — from both SpV (Fig. 3) and skin mucus — as a 14 kDa band in SDS-PAGE. Analytical gel filtration on a calibrated size exclusion column provided several peaks, most of which contained this same protein in different oligomeric states (mainly as a tetramer). Cross-linkage studies confirmed the oligomeric nature of this integrin-inhibiting factor. Plumieribetin is characterized by an abundance of anti-parallel beta strands, just as the aforementioned B-type lectins. The primary structure of plumieribetin is highly similar to those of homologous proteins isolated from other fishes, namely *Platycephalus indicus* (71.5%), the green puffer fish *Tetraodon nigroviridis* (63.7%) and the Japanese pufferfish *T. rubripes* (56.8%) [27].

Plumieribetin binds to α1β1 integrin irrespective of N-glycosilation — indicating direct protein-protein interaction — supressing α1β1 integrin binding to basement membrane collagen IV. It could not fully detach hepatocarcinoma HepG2 cells or primary arterial smooth muscle cells from collagen IV fragment CB3. It did, however, attenuate cell-collagen contacts and cell spreading, changing the actin cytoskeleton after blocking the compensating α2β1 integrin as well [27].

In addition to the hemagglutinating fraction (FV) (Fig. 3), five main absorbance peaks were detected by reverse phase high performance liquid chromatography (RP-HPLC) (RP1, 2, 3, 4 and 5). Mass spectrometry analysis of these fractions on matrix-assisted laser desorption/ionization — time of flight (MALDI-TOF) revealed a high degree of homogeneity with m/z signals and molecular masses of 16.981, 16.982, 16.975, 16.841 and 16.842 kDa. The amino acid sequence of RP4 revealed homology (24–32% of identity) with various fish C-type lectins. Finally, the presence of the glycan moiety galactose-β(1 → 4)-N-acetylglucosamine was also revealed in the FV structure [28].

The similar chemical characteristics exhibited by RP fractions (elution in RP-HPLC and MALDI-TOF) — together with the similarities found among amino acid sequences — strongly suggest that RP1-5 are C-type lectin isoforms (isolectins) [28].

### *Scorpaena plumieri* cytolytictoxin (SP-Ctx)

Considerable evidence suggests that the cardiovascular, inflammatory and cytolytic effects attributed to Scorpaenidae fish venoms are due to the action of a single labile “lethal protein factor” [1, 5, 56].

A cytolysin denominated Sp-CTx — a glycoprotein with two subunits (of ≈ 65 kDa each) — was purified from the venom of *S. plumieri* [24]. Next, an improved purification approach was established, which reduced the time and the number of chromatography steps needed to obtain the pure toxin [25]. Due to the lability of Sp-CTx, such a time reduction is crucial to the success of its isolation and functional characterization.

**Fig. 4:**
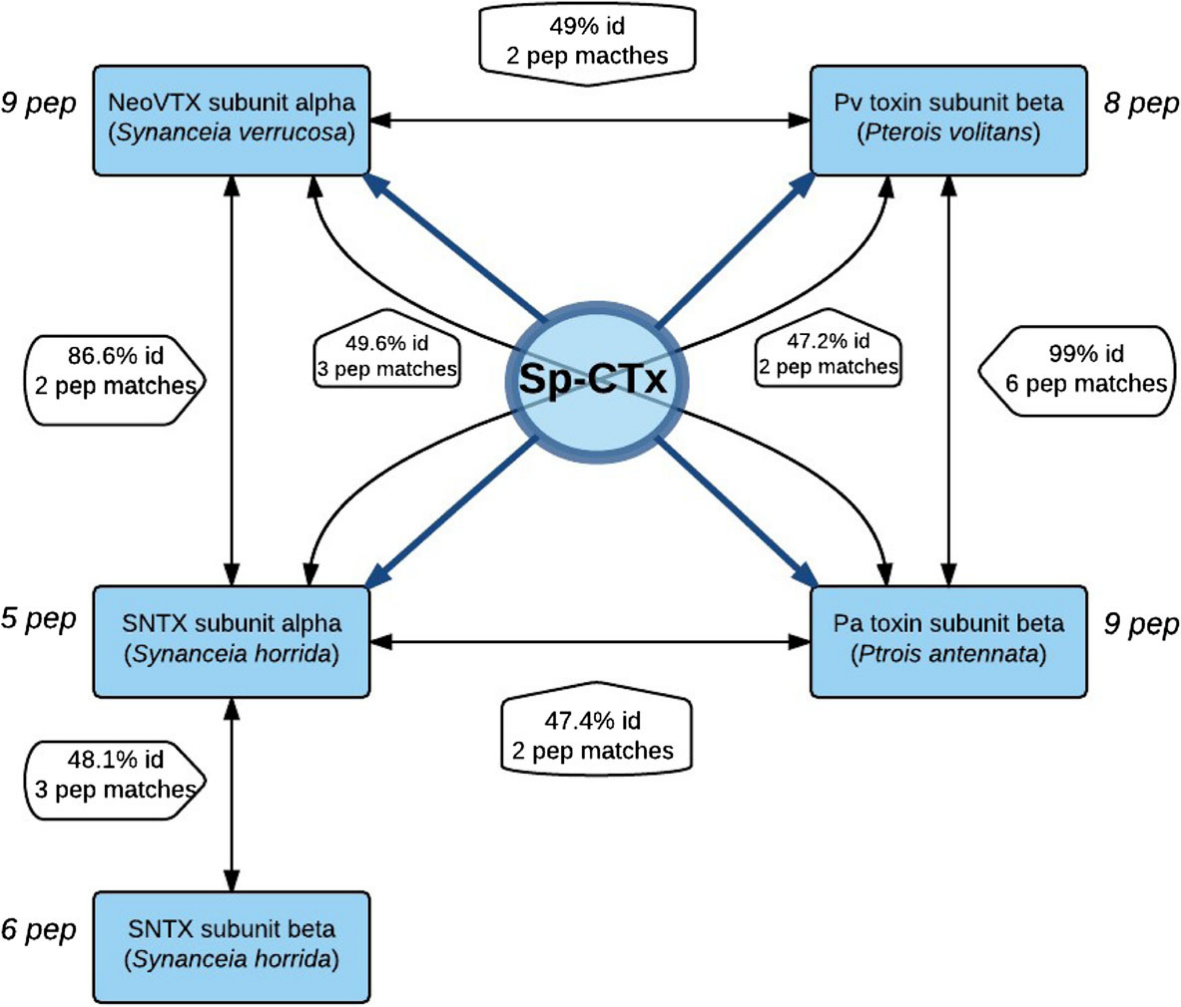
Identity percentage (coverage id) between fish toxins (blue boxes). The number of Sp-CTx-predicted tryptic peptides (pep “matches”) shared between corresponding subunits is represented along with the respective percentage values. The “pep” values depicted beside each blue box stand for the number of Sp-CTx-predicted peptide fragments that are shared with each appointed subunit

Orbitrap-MS analyses revealed thirty-seven Sp-CTx internal amino acid sequences after proteolytic fragmentation with trypsin. Through the protein database NCBInr, 29 tryptic peptide fragments were found to have identity with other oligomeric cytolysins (SNTX, neoVTX, Pvtoxin or/and Patoxin, Fig. 4) from fishes belonging to the families Scorpaenidae and Synanceiidae [25]. The evolutionary implications of this similarity reinforces the idea of a close relationship between scorpionfish, lionfish and stonefish, already suggested based on phylogeny studies [6].

Like other fish cytolysins, Sp-CTx has shown hemolytic activity in rabbit erythrocytes attenuated by osmotic protectants (polyethylene glycol polymers) and molecules larger than 6 nm in diameter. This strongly suggested that Sp-CTx might be a pore-forming protein, since it lacks phospholipase A2 activity [25]. Furthermore, previous reports have shown that the hemolytic effect induced by SNTX was fully prevented by osmotic protectants of adequate size while uncharged molecules of smaller size failed to avert cell lysis [57]. More recently, the pore formation mechanism was directly visualized through transmission electron microscopy of SNTX [58].

Despite its hemolytic effect, the focus of Sp-CTx research has been on its cardiovascular activities. In vivo and in vitro (isolated hearts) studies revealed that Sp-CTx reproduces the effects induced by SpV. In isolated papillary muscle, Sp-CTx produces a positive inotropic effect attenuated by propranolol and the catecholamine releasing agent tyramine, while increasing L-type Ca^2+^ current density in isolated ventricular cardiomyocytes. These results show that Sp-CTx induces cardiovascular disorders through an increase of sarcolemmal calcium influx, partially due to the release of endogenous noradrenaline [26]. In addition, Sp-CTx reproduced the SpV-induced effect on aortic rings, although the relaxation phase is less marked in this case. This relaxant effect is abolished after endothelial denudation, suggesting that the release of endothelium-derived relaxing factors is involved in this response [24].

Besides the cytolytic and cardiovascular effects displayed by cytolysins isolated from fish venoms, other pharmacological effects such as edematogenic and nociceptive activities have been reported [1]. As to Sp-CTx, a thorough investigation of its role in the inflammatory effect induced by SpV remains to be done.

A summary of the bioactive proteins isolated from SpV is presented below (Table 1), along with their chemical and functional features.

**Table 1:** Toxins purified from SpV to date

Molecule Function	Toxin Name	Chemical Aspects	Functional Characteristics	Source
Protease	Sp-GP	≈72 kDa N-terminally blocked	Gelatinolytic activity	[20]
Cytolysin	Sp-CTx	Glycoprotein Dimeric constitution (subunits ≈ 65 kDa)	Hemolytic activity — cell membrane pore formation Cardiovascular biphasic response in vivo — initial systolic and diastolic pressure increase followed by decrease Positive inotropic effect on cardiac muscle Increase of Ca^2+^ current on isolated cardiomyocytes Vasoconstriction — coronary bed Vasodilation — aortic ring	[24] [25] [26]
B-type lectin	Plumeribetin	Homotetramer (monomer — 13.157 kDa) N-terminally blocked High content of anti-parallel strands	Integrin inhibitory activity Attenuation of cell-collagen contacts and cell spreading	[27]
C-Type lectin	Sp-LC 1 2 3 4 5	16.981 kDa 16.982 kDa 16.975 kDa 16.841 kDa 16.842 kDa	Hemagglutinating activity Recognizes the sugar motif (Gal-β(1 → 4)GlcNAc)	[28]

## Molecular genetics of *S. plumieri* venom

The difficulties surrounding the study of fish venoms also affect their characterization at the molecular level. To date few reports have been published regarding the analysis of fish venoms from a genetic point of view [59–62]. Transcriptomic approaches performed on the venom glands of the stingray *Neotrygon kuhlii* [60] and the toadfish *Thalassophryne nattereri* [59] revealed a considerable number of proteins that are related to the pharmacological activity of these venoms — e.g. galectins [60] and C-type lectins [59] — as well as some that are novel to fish venoms. A preliminary analysis of expressed sequence tags (EST) obtained through a cDNA library from *S. plumieri* venom revealed that about 30% of the sequences had no similarities with previously described ones, suggesting the presence of unknown genes of potential relevance in the venom gland. In addition, the screening of the library with antibodies against a lectin fraction from *S. plumieri* venom has shown that lectin-like genes account for 12% of all transcripts, a finding confirmed by extensive *in silico* analysis [61]. These constitute the very first steps towards the unraveling of the molecular diversity contained in fish venoms.

## Neutralization of *S. plumieri* toxic activities

Although there is no antivenom available for the envenoming by *S. plumieri*, the commercial antivenom raised against the venom of the stonefish *Synanceia trachynis* (SFAV) — a horseFab’2 preparation made by CSL in Melbourne, Australia [63] — evoked a cross-reactive immune response to SpV.

SFAV neutralizes all known clinical effects of serious *S. trachynis* envenomation [64], and is also efficient in neutralising the inflammatory and cardiovascular responses as as well as the hemolytic activity induced by *S. plumieri* in mice [29], suggesting that the compounds responsible for these effects share similar biochemical and antigenic properties to those found in stonefish venom. This antivenom also neutralises some of the toxic effects of other stonefish (*S. verrucosa*), lionfish (*Pterois volitans, P. lunulata, P. antennata and Dendrochirus zebra*) and soldierfish (*Gymnapistes marmoratus*) [51, 65, 66].

This is in accordance with the hypothesis that venomous fishes belonging to different genera or inhabiting different regions may share venom compounds with similar antigenic properties [1].

## Conclusions

In conclusion, despite all the progress made recently, many questions remain to be answered, not only with respect to the physio-pharmacological effects and the precise action mechanism of some of the components already described, but also as to the considerable number of molecules still unexplored in the venom of *S. plumieri*. The study and exploration of the full potential contained in fish venoms can contribute to a better understanding of complex physiological processes — such as the very pain induced by the envenomation — and to the discovery of new drugs, not to mention the development of more effective ways to treat the injuries caused by these animals.

## Abbreviations

AM: Alveolar macrophages
CPP: Coronary perfusion pressure
ECM: Extracellular matrix
EST: Expressed sequence tags
MALDI-TOF: Matrix-assisted laser desorption/ionization – time of flight
MAP: Mean arterial pressure
RP-HPLC: Reverse phase high performance liquid chromatography
SINAN: Notifiable diseases information system
Sp-GP: *Scorpaena plumieri* gelatinolytic protease
SpV: *S. plumieri* venom extract
UFES: Federal University of espírito santo
